# Hypoxia, metabolism, and the circadian clock: new links to overcome radiation resistance in high-grade gliomas

**DOI:** 10.1186/s13046-020-01639-2

**Published:** 2020-07-07

**Authors:** Han Shen, Kristina Cook, Harriet E. Gee, Eric Hau

**Affiliations:** 1 Translational Radiation Biology and Oncology Laboratory, Centre for Cancer Research, Westmead Institute for Medical Research, Westmead, New South Wales 2145 Australia; 2 Sydney Medical School, University of Sydney, Camperdown, New South Wales Australia; 3 Faculty of Medicine and Health & Charles Perkins Centre, University of Sydney, Camperdown, New South Wales Australia; 4 Department of Radiation Oncology, Crown Princess Mary Cancer Centre, Westmead Hospital, Westmead, New South Wales Australia; 5 Blacktown Hematology and Cancer Centre, Blacktown Hospital, Blacktown, New South Wales Australia

**Keywords:** Radiotherapy, Hypoxia, Circadian rhythm, High-grade glioma, Metabolism

## Abstract

Radiotherapy is the cornerstone of treatment of high-grade gliomas (HGGs). It eradicates tumor cells by inducing oxidative stress and subsequent DNA damage. Unfortunately, almost all HGGs recur locally within several months secondary to radioresistance with intricate molecular mechanisms. Therefore, unravelling specific underlying mechanisms of radioresistance is critical to elucidating novel strategies to improve the radiosensitivity of tumor cells, and enhance the efficacy of radiotherapy. This review addresses our current understanding of how hypoxia and the hypoxia-inducible factor 1 (HIF-1) signaling pathway have a profound impact on the response of HGGs to radiotherapy. In addition, intriguing links between hypoxic signaling, circadian rhythms and cell metabolism have been recently discovered, which may provide insights into our fundamental understanding of radioresistance. Cellular pathways involved in the hypoxic response, DNA repair and metabolism can fluctuate over 24-h periods due to circadian regulation. These oscillatory patterns may have consequences for tumor radioresistance. Timing radiotherapy for specific times of the day (chronoradiotherapy) could be beneficial in patients with HGGs and will be discussed.

## Background

HGGs are diagnosed in patients of all ages, including children. Glioblastoma, the most aggressive HGG, typically occurs between 45 and 75 years of age and carries a dismal prognosis of less than 15 months. Surgery and radiotherapy have been the cornerstone of treatment for glioblastoma. To improve the poor outcomes associated with this disease, numerous therapeutics have been added to radiotherapy without success until the landmark study established a standard of care with gross surgical excision followed by concurrent temozolomide and radiotherapy [1]. While gains have been made in understanding glioblastoma biology, improving patient outcome remains a significant challenge. Although the addition of temozolomide has modest overall survival benefit in patients with methylated *MGMT* promoter, it confers little benefit in the 60% of glioblastoma patients with unmethylated *MGMT* promoter [2]. In children, the incidence of HGGs is much lower, but they similarly have a very poor prognosis overall, with treatment consisting of maximal surgical resection followed by radiotherapy. The role of temozolomide is less clear in pediatric high-grade gliomas (pHGGs) as compared to glioblastoma. Among all the pHGGs, diffuse intrinsic pontine glioma (DIPG) is the most aggressive form arising from the centre of the brainstem, the most critical area of the brain. As a result, surgical resection is impossible, and in most cases even a biopsy is hazardous. During the past decade, clinical trials of chemotherapeutic agents and targeted therapies in DIPG have not shown any survival benefit, and radiotherapy is the only standard treatment [3].

**Fig. 1:**
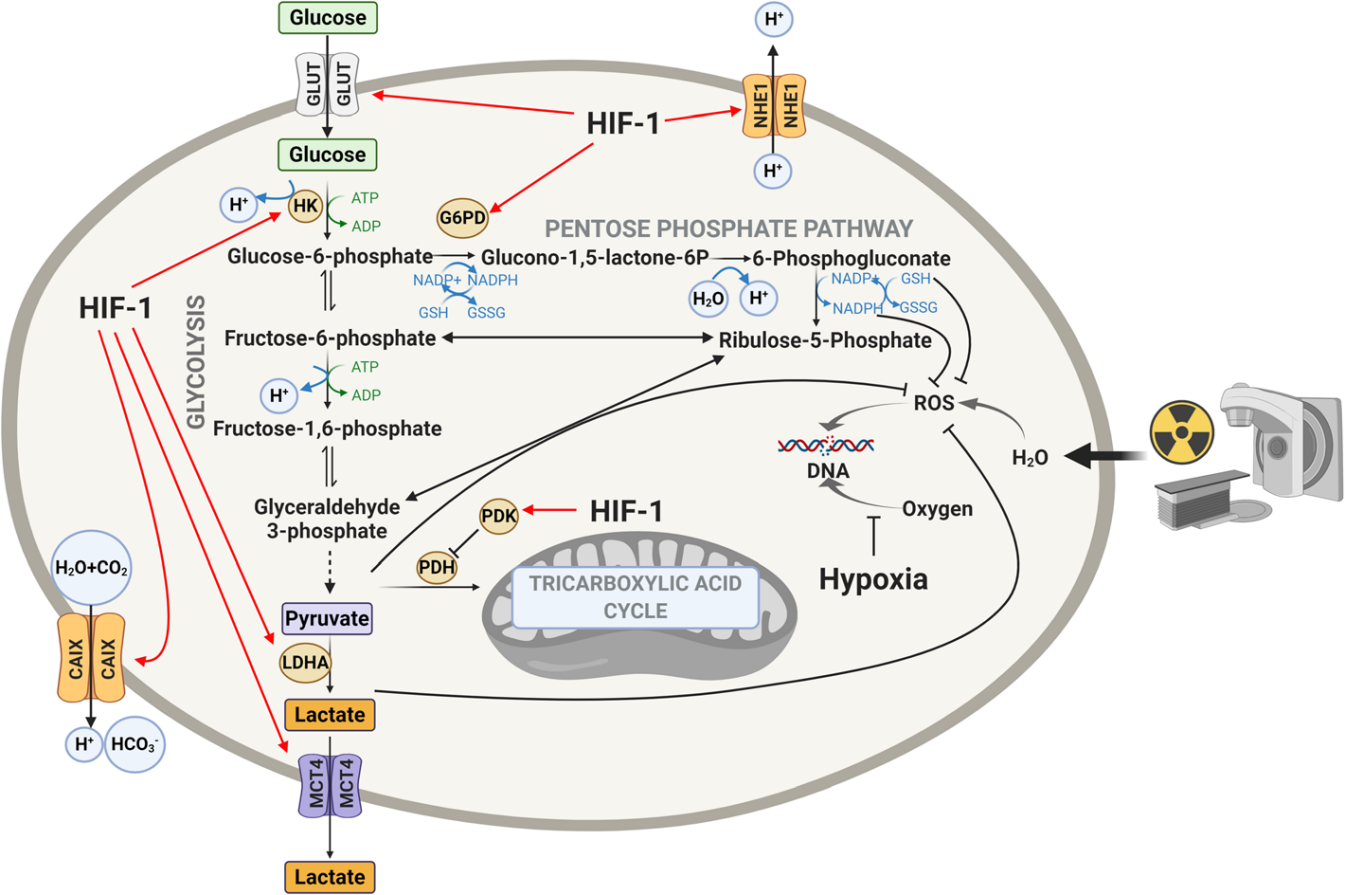
The mechanism of actions whereby the hypoxia inducible factor (HIF-1) regulates glycolysis, lactate pathway and pentose phosphate pathways in radioresistance. ADP, adenosine diphosphate; ATP,adenosine triphosphate; CAIX, carbonic anhydrase 9; G6PD,glucose-6-phosphate dehydrogenase; GLUT, glucose transporters; GSH, glutathione; GSSG, glutathione disulfide; HIF-1, hypoxia inducible factor-1; HK, hexokinase; LDHA, lactate dehydrogenase A; MCT4, monocarboxylate transporter 4; NHE1, Na+/H+ exchanger isoform 1; PDH, pyruvate dehydrogenase; PDK, pyruvate dehydrogenase kinase; ROS, reactive oxygen species

**Fig. 2:**
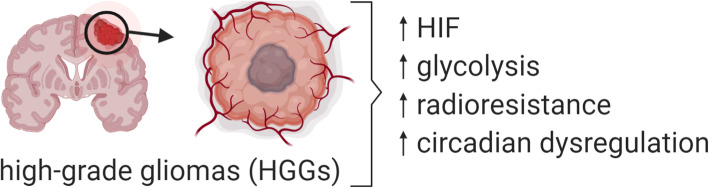
High-grade gliomas (HGGs) have multiple characteristics that contribute to their aggressive behavior. Increased levels of HIF, glycolysis and radioresistance are common. Circadian dysregulation has also been identified as a recent characteristic contributing to growth, stemness and metabolic changes observed in HGGs

Radiotherapy is the targeted administration of X-rays to destroy cancer cells and tumor tissue. It targets rapidly proliferating tumor cells by inducing oxidative stress through increased Reactive Oxygen Species (ROS). With active oxygen molecules including superoxide and hydroxyl radicals, ROS break chemical bonds and activate the cascade in DNA damage and subsequent cell death. During this process, oxygen is critical to stabilize the DNA damage induced by ROS, and this forms the basic mechanism by which radiation is used in cancer treatment [4]. Hypoxia, a common characteristic of most solid tumors, prevents the fixation of DNA damage by oxygen, thus being a major cause of radioresistance in cancer treatment [5]. Furthermore, hypoxia activates the hypoxia-inducible factor 1 (HIF-1) pathway which favors the survival of tumor cells by increasing their glucose uptake and utilization via altered glucose metabolism [6]. It also induces angiogenesis [7], creates an acidic microenvironment and promotes proliferation [8], all of which collectively dampen the efficacy of radiotherapy (Fig. 1). HIF-1 is also well known to be expressed constitutively in cancer cells under normoxic conditions through cancer-specific genetic alterations [9]. Radiotherapy, on the other hand, has also been reported to stabilize HIF-1 signaling via radiation-induced reoxygenation, ROS elevation and microvascular destruction, leading to the development of acquired radioresistance [10]. Interestingly, the dysregulation of circadian rhythm has also recently been identified as a characteristic contributing to growth, stemness and metabolic changes observed in HGGs [11] (Fig. 2). The circadian rhythm disorder modulated by HIF-1 signaling may even further affect the radiosensitivity of tumor cells. In this review, we summarize and discuss how the HIF-1 pathway impacts on radioresistance of solid tumors with a focus on HGGs, via modifying glucose metabolism and circadian rhythm of tumor cells. Furthermore, inhibition of HIF-1 modulated glucose metabolism and circadian rhythm is also discussed as a potential approach to overcoming radioresistance of HGGs in both adult and pediatric settings.

### Radioresistance of HGG cells

Irradiation as a cancer therapy, was discovered over twelve decades ago and has become the cornerstone of treatment for many types of cancer. For HGGs, radiotherapy is either given as definitive treatment for inoperable tumors or delivered post-surgery to the area of the excision to kill residual tumor cells. The effectiveness of radiotherapy relies mainly on its ability to cause lethal damage to the DNA of cancer cells, with contributions from damage to sub-cellular organelles, and triggers cell death by inducing cellular stress response and activating intracellular signaling pathways resulting in cell death [12]. Although radiotherapy is one of the most effective therapies for cancer treatment, most malignant tumors inevitably relapse secondary to radioresistance. This is particularly true for HGGs due to the high intrinsic radioresistance of glioma cells, especially in the presence of glioma stem cells (GSCs) that are enriched in the hypoxic niche [13]. Radioresistance is a process in which either the intrinsically radioresistant cells are selected for by radiotherapy or the surviving tumor cells adapt and develop acquired resistance due to the radiotherapy-induced changes. This is an intricate process involving multiple mechanisms which remain to be fully elucidated. Hitherto, both pre-clinical and clinical studies have revealed some of the mechanisms underlying this phenomenon, including changes in capability of DNA repair, cell cycle arrest, alterations of gene expression, changes in microenvironment, induction of autophagy, generation of cancer stem cells, and rewiring of metabolic pathways [14–16]. In the following section, we will primarily focus on metabolic reprogramming, one of the hallmarks of cancer, because most of those aforementioned machineries converge in a common adaptation of tumor cell metabolism that is strongly linked to radioresistance in cancer treatment.

#### Role of hypoxia, HIF signaling mediated glucose metabolism and radiotherapy in radioresistance of HGGs

##### Hypoxia in HGGs

Hypoxia, or physiologically low levels of oxygen tension, develops in HGGs from uncontrollable cell proliferation and dysfunctional vascularization. In patients with a malignant solid tumor, hypoxia is strongly correlated with a poor prognosis, an increased chance of metastasis and resistance to chemoradiotherapy [17]. It is generally accepted that the oxygen level in hypoxic tumor tissues is much lower than that in their corresponding normal tissues and on average it is below 1–2% O_2_ (v/v) [18]. In glioblastoma, the hypercellular zones, referred to as ‘pseudopalisading necrosis’, typically surrounding necrotic foci are constantly exposed to moderate hypoxia [19]. Hypoxia in glioblastoma has been observed by magnetic resonance imaging (MRI) where reduced oxygen diffusion is detected, consistent with restricted blood flow [20]. Molecular markers of hypoxia, such as HIF-1 and its downstream targets vascular endothelial growth factor (VEGF) and carbonic anhydrase 9 (CA IX), are consistently detected in glioblastoma using immunohistochemistry staining [21], while dynamic contrast enhanced MRI reveals tumor vascularity [22]. These features correlate with worse progression-free and overall survival [23, 24]. Although hypoxia is a well-known characteristic in adult HGGs, its role in pHGGs remains relatively less known. Genomics and metabolomics data from a recent study support hypoxia as an important key to preserve the abnormal metabolism of pHGG cells and cell dissemination present in pHGG patients [25]. In addition, DIPGs in the brainstem have been detected with low blood perfusion and regions of necrosis by using MR spectroscopy, indicative of a hypoxic environment that supports activation of HIF, which is associated with increased proliferation, invasion and therapy resistance [26].

##### HIF-1α mediated reprogramming of glucose metabolism and radioresistance

Hypoxia induces transcriptomic changes followed by proteomic alteration within tumor cells. At the molecular level, the adaptation of tumor cells to reduced oxygen tension is regulated mainly by HIF, a transcription factor which stabilizes in response to reduced oxygen levels. In the human HIF family, there are three isoforms, HIF-1, HIF-2 and HIF-3, all of which are heterodimers comprising of α and β subunits. Of these three members, HIF-1α is frequently overexpressed in tumor cells, and is the most extensively studied in cancer research. HIF-1α is ubiquitously expressed throughout tissues in the body, while the HIF-2α and HIF-3α isoforms are only expressed in select tissues (including the brain) and are not as well studied.

The transcription of genes that encode glucose transporters and enzymes regulating glycolysis and the pentose phosphate pathway (PPP) is extensively regulated by HIF-1α [27] (Fig. 1). As hypoxia suppresses the activities of the key enzymes controlling the rate of tricarboxylic acid cycle, tumor cells switch to glycolysis for energy production [28]. Pyruvate, lactate, and hydrogen ions are abundantly produced in anaerobic glycolysis or the Pasteur effect [29]. In contrast to the Pasteur effect, a hallmark of cancer cells is a high rate of glucose consumption and lactate generation even in the presence of ample oxygen. This distinctive metabolic feature of cancer cells was first observed in the 1920s by Otto Warburg [30], referred to as the Warburg effect. With the Warburg effect, cancer cells heavily rely on the glycolytic pathway to support their energy demands under normoxia [31]. This is also supported by later studies showing the constitutive expression of HIF-1α in many cancer cell lines independent of hypoxic circumstances [32]. Although the glycolytic phenotype may seem counterintuitive due to the less efficient ATP production by glycolysis compared with oxidative phosphorylation, studies have revealed the use of glycolysis by cancer cells provides a growth advantage and facilitates malignant progression [31]. For example, the glycolytic intermediate glucose-6-phosphate (G-6-P) is also used in the PPP, which synthesizes precursors of nucleotides and amino acids required for tumor cell growth and proliferation.

HGGs, like most malignant solid tumors, are highly glycolytic, producing large amounts of lactate as a metabolic by-product [33]. It has been shown that tumors with high levels of glycolysis are less responsive to chemoradiotherapy and behave more aggressively [34]. More recent reports have also identified the Warburg effect to be implicated in resistance to oxidative stress induced by radiotherapy [35]. Since G-6-P serves as a substrate for the PPP responsible for the biogenesis of the antioxidants NADPH and glutathione [36], the HIF-1α–mediated Warburg effect has been associated with the increased antioxidant capacity of tumor cells and eventual radioresistance [37]. A very recent study reported TP53 mutation as the main driver of increased radioresistance in both patients and corresponding cellular models of DIPG [38]. TP53 is known to impact glycolysis through several mechanisms including transcriptionally repressing the expression of glucose transporters, down-regulating rate limiting enzymes of glycolysis, decreasing the expression of transporters responsible for lactate extrusion and negatively regulating the AKT/mTOR [39] and NF-κB signaling pathways [40]. It can also modulate expression of glycolytic enzymes like phosphoglycerate mutase and TP53-induced glycolysis and apoptosis regulator (TIGAR), a stimulator of gluconeogenesis and the PPP [41]. These findings collectively indicate the aberrant glucose metabolism may also play an important role in radioresistance of DIPG and other pHGGs under the influence of TP53 mutation.

##### Acquired radioresistance induced by radiotherapy

Although radiotherapy is used to eradicate any remnant tumor cells after surgery, radiation is ironically known to transform tumor cells into a more aggressive and radioresistant form, for which the treatment options are very limited [42]. Several mechanisms have been proposed for this paradoxical phenomenon. Firstly, radiotherapy-induced reoxygenation can stabilize HIF-1 signaling and this is believed to be involved in radioresistance. Radiation is delivered in multiple dose fractions to enable tumor reoxygenation in-between fractions [43]. Tumor shrinkage over the course of radiotherapy allows blood vessels to reach previously distant cells, thus reducing the hypoxic fraction and reoxygenating hypoxic cells. Following delivery of radiotherapy, reoxygenation and a temporary reduction in hypoxia occurs due to the aerobic tumor cells being killed. However, the radiotherapy-induced reoxygenation also elevates the production of ROS, induces depolymerization of cytoplasmic stress granules containing HIF-1–regulated mRNA transcripts, and activates PI3K/AKT/mTOR pathway, all of which eventually stabilizes the HIF-1 expression in surviving tumor cells [44]. Secondly, the post-radiotherapy upregulation of genes controlling DNA repair, cell proliferation and anti-apoptosis is also believed to play an important role in radiotherapy-induced radioresistance, particularly when a sub-lethal dose of radiotherapy was cumulatively delivered [45]. This has been frequently reported by studies investigating adult glioblastomas and a very recent report has also confirmed the similar findings in pHGGs [46]. Last but not least, radiotherapy-induced generation and enrichment of cancer stem cells is also a major obstacle for maintaining the efficacy of radiotherapy to eradicate cancer cells. Bao et al. reported for the first time that the percentage of CD133-positive cells increased in gliomas after radiotherapy, indicative of an important role of radiotherapy itself in the development of radioresistance [47]. In addition, recent studies have consistently reported that radiotherapy enhances the transdifferentiation of glioma stem-like cells into tumor derived endothelial cells which may further contribute to neovasculature and radioresistance [48, 49]. In the end, the surviving radioresistant tumor cells will eventually proliferate and lead to cancer relapse within the previously irradiated field, which challenges the role of re-irradiation in the treatment of recurrent HGGs.

#### Role of hypoxia and circadian rhythm in radioresistance of HGGs

Circadian rhythms are endogenous, 24-h cycles that regulate physiological, behavioral and cellular changes in organisms. These rhythms control pathological processes that contribute to HGGs, including those involving cell cycle, immune surveillance, metabolism and more [50, 51]. Radiotherapy induced DNA damage has also been shown to be regulated by circadian rhythms [52]. Furthermore, GSCs depend on circadian rhythms for growth, metabolism and stemness [11], all of which impact the efficacy of radiotherapy. A limited number of studies have shown that HIF pathways interact with the circadian pathways though this is yet to be investigated in HGG radiotherapy models.

##### The biology of circadian rhythms and the molecular clock

**Fig. 3:**
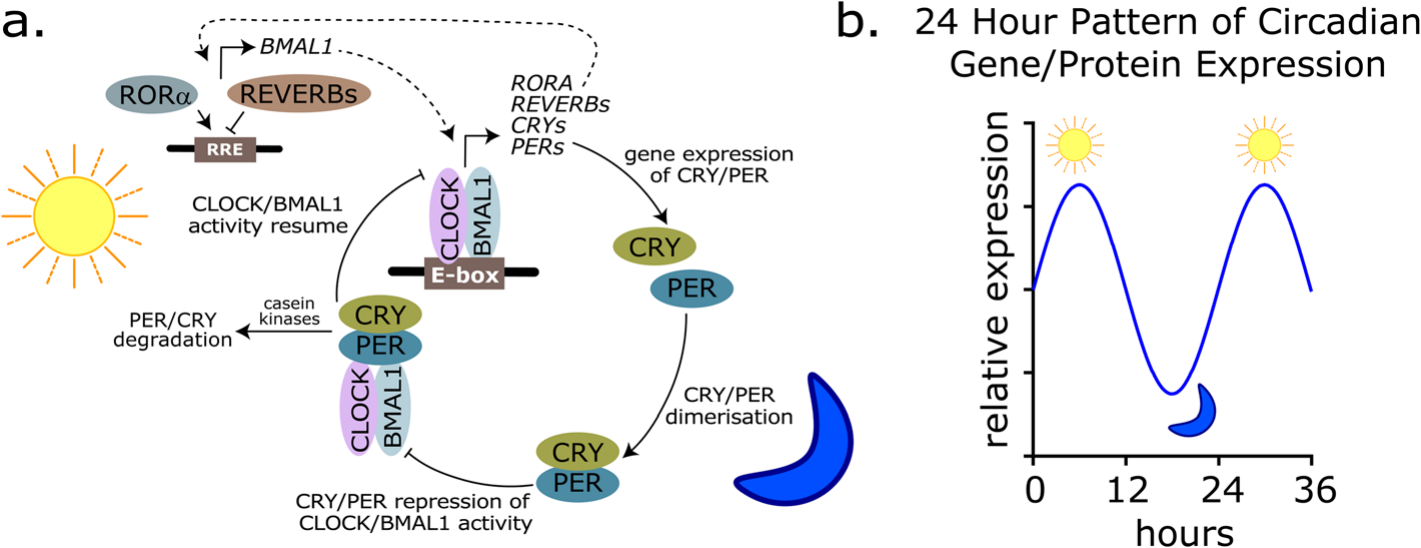
**a** The basic mammalian circadian clock loop consists of transcription factors BMAL1 and CLOCK which express PER (PER1, PER2, PER3), CRY (CRY1, CRY2), REV-ERB (NR1D1, NR1D2) and ROR (RORA, RORB, RORC) genes. The PER and CRY proteins accumulate and repress BMAL1/CLOCK activity before being degraded through a mechanism involving casein kinases and the proteasome, which allows BMAL1 and CLOCK activity to resume. In a second loop, the expressed ROR and REV-ERB proteins act to enhance or repress expression of BMAL1 gene. Genes/mRNAs indicated in *italics*, proteins indicated by colored ovals. **b** Circadian genes demonstrate cyclical expression over a 24 *h* period. BMAL1, brain and muscle aryl hydrocarbon receptor nuclear translocator (ARNT)-like 1; CLOCK, circadian locomotor output cycles kaput; PER, period; ROR, RAR-related orphan receptor

The circadian machinery is disrupted in a wide range of cancers [53], including HGGs [11, 54–56], and it appears to have context-dependent roles that contribute to malignant behavior and radiotherapy resistance. Circadian rhythms are generated by a molecular clock encoded in the genome, which consists of a network of interlocking transcriptional-translational feedback loops (Fig. 3a). The central feedback loop is composed of transcription factors, BMAL1 and CLOCK, which dimerize and bind to the E-box DNA sequence in the promoters of clock-controlled genes, including the period (PER) and cryptochrome (CRY) genes. CLOCK/BMAL1 induces the expression of PER and CRY isoforms, which act as negative regulators of CLOCK/BMAL1 activity. PER and CRY proteins accumulate, before being degraded by the proteasome through pathways including the casein kinases. This enables CLOCK/BMAL1 activity to resume, repeating the cycle approximately every 24 h. There are also additional feedback loops involving RORα and REV-ERBs, which act to increase or repress the expression of BMAL1, respectively [50]. Circadian rhythms are maintained in nearly every cell of the body and they are cell autonomous, but individual cells are synchronized to one another by external cues, such as light, food intake and temperature. Epidemiological studies have shown that environmental disruption of circadian rhythms may increase the risk of developing some cancers [57–59]. Genetic disruption of circadian clocks in animals can also promote tumorigenesis and progression of specific cancers [52, 60–62].

##### Studies on circadian rhythms and gliomas

There are limited studies on circadian rhythms specifically in HGGs. Abnormal rhythms are associated with high-grade brain tumors [54, 63] and HGGs may have different cellular circadian rhythms when compared to surrounding healthy tissues [55, 56]. These altered rhythms can result from genetic variations, chromosomal amplifications and/or changes to gene expression in clock-related genes. For example, a genetic variant of PER1 has been associated with overall glioma risk [64], and decreased PER1 mRNA and protein has been observed in HGGs when compared to lower-grade gliomas (LGG) [65]. REV-ERBβ (NR1D2) is highly expressed in glioblastoma, and is required for cell proliferation, migration and invasion [66]. Expression of CRY1 and CRY2 is lower in HGGs compared to surrounding non-glioma cells [67]. Chronic jetlag, an environmental circadian disruption, leads to differential regulation of glioma-related genes in the brain of mice with and without a functioning clock. These results indicate that the molecular clock could potentially play a role in glioma risk [68].

The CLOCK gene is amplified in ~ 5–9% of glioblastoma patients [69, 70]. In support of this, several studies have found that CLOCK expression is increased in HGGs when compared to LGG or non-malignant cells [54, 71]. CLOCK and its binding partner, BMAL1, are thought to contribute to glioma proliferation, migration, stemness and metabolic reprogramming [11, 66, 70, 71] and a recent paper proposed that GSCs use BMAL1 and CLOCK to alter these characteristics and gain a physiological advantage [11]. CLOCK and BMAL1 knockdown decreased glioblastoma proliferation through cell-cycle arrest and apoptosis. This effect was especially potent in GSCs when compared to differentiated glioblastoma cells. Normal neural stem cell growth was unaffected by CLOCK and BMAL1 knockdown, indicating that glioblastoma cells are unique in relying on these circadian transcription factors for maximum cell growth [11].

Taken together, these studies support the emerging idea that BMAL1 and CLOCK have tumor promoting qualities in HGGs, while PER (and possibly CRY) may have tumor suppressing roles, though this is likely to be context and cancer dependent. A pan-cancer study using genomic, transcriptomic and clinical data largely supports this [55]. Downregulation of the circadian genes encoding CRY2, PER1, PER2, PER3, CLOCK, REV-ERBβ, ROR-α and ROR-β was associated with significantly higher mortality rates in the glioma cohort, hinting at possible tumor suppressor roles. Overexpression of BMAL1 in gliomas led to a poorer survival outcome [55], indicating a potential tumor promoting role. Interestingly, in this study, CLOCK downregulation in gliomas was associated with higher mortality [55] which conflicts with other studies where CLOCK is overexpressed [54] or amplified [69, 70] in HGGs and contributes to malignant changes [11]. However, this study was limited by a small number of non-tumor brain samples (five), which were used as a comparison to determine over- and under-expression [55]. Further study is clearly warranted but overall circadian changes were strongly linked to gliomas [55].

##### Links between circadian disruption, tumor hypoxia and HIF

One interesting finding to emerge from this largescale multi-cancer bioinformatics analysis was an association between markers of tumor hypoxia and circadian dysregulation, particularly in gliomas [55]. The loss of proposed circadian tumor suppressors and/or an increase in circadian tumor promoters was associated with markers of tumor hypoxia, leading the authors to propose that circadian dysregulation is exacerbated by hypoxia [55]. There is evidence that hypoxia and HIF can alter circadian rhythms and vice versa in a non-tumor context [72–75]. This bidirectional relationship may be important in HGGs, as both hypoxia and circadian disruption can play a role in tumor progression. While these relationships have only been studied in specific cell types and under certain circumstances, these studies provide justification for exploring in the context of HGGs.

Circadian gene expression is altered by hypoxia in hepatocellular carcinoma cells through a mechanism dependent on HIF-1α [76]. PER1 and CLOCK levels increase in the brain when mice are exposed to hypoxia [77]. These observations have led to several proposed mechanisms, some of which have evidence in vitro and in vivo. Hypoxia and HIF-1α can directly alter and regulate the molecular clock through HIF-1α binding to BMAL1 to express E-box circadian genes, including the PER and CRY genes in vitro [78]. Overexpression and stabilization of HIF-1α increases expression levels of circadian genes [73, 75]. This may mean that hypoxia could lead to the expression of circadian genes at inappropriate times. HIF-1α can also alter the molecular clock through indirect effects. HIF controls genes involved in the production of acid, and an acidic environment suppresses oscillation of the molecular clock by inhibiting mTORC1 [79].

Circadian rhythmicity also can affect the HIF-1α hypoxic response. HIF-1 activity is gated by the circadian clock, meaning that the strength of the hypoxic response is clock-regulated [73]. Furthermore, there is an E-box in the promoter of the *HIF1A* gene, indicating that CLOCK/BMAL1 can induce expression of *HIF1A* [73]. CRY1 can bind directly to the HIF-1α protein in mice, which dampens HIF-1 expression of target genes [80]. This means that during periods of high CRY1 expression, the maximal HIF-1α hypoxic response can be dampened, though still present. During periods of low CRY1, HIF activity increases, which leads to cellular proliferation and migration in some circumstances [80]. Furthermore, PER2 has been found to bind to HIF-1α, which increases HIF-1 activity, and has the opposite effect of CRY2 [81].

Synergistic effects have also been observed between HIF and CLOCK/BMAL1. Both heterodimer transcription factors co-occupy core clock genes, including PER1 and PER2, and it is thought that this is due to both E-box and hypoxia response element (HRE) motifs being present in the promoter of the same gene [73]. The consequences of these findings for HGGs is difficult to interpret, as most of these studies were conducted in tissues other than the brain and not in gliomas. However, circadian rhythms, hypoxia and HIF-1 have all been shown to contribute to enhance progression in HGGs and should be investigated further.

##### Circadian rhythms and radiotherapy

DNA repair genes have cyclical expression patterns in mRNA and protein expression [82, 83] and there is some initial evidence that these rhythms can influence radiosensitivity. Radioresistance is highest in the second half of the daily light span when mice are exposed to whole body irradiation [84]. When the same mice were exposed to a jet-lag protocol ‘shifting their time of day’, radiation sickness was worsened. Further studies have begun to unravel some of the possible reasons for circadian-regulated radiosensitivity. Nucleotide excision repair by xeroderma pigmentosum, a DNA damage recognition protein, peaks in the afternoon/evening and is at its lowest in the night/early morning in the mouse brain cortex [85] and the skin [86]. These effects can be seen in glioma models, as rat glioma cells synchronize at the G2/M transition of the cell cycle during periods of high PER2 expression, and this makes them more sensitive to radiotherapy [87, 88]. PER1, another period isoform, may have a role in radiation-induced apoptosis and DNA damage in gliomas, as downregulation of PER1 attenuated DNA damage in U343 glioma cells [89]. This may have consequences for glioma treatment as decreased levels of PER1 have been observed in HGGs [65] and this may make tumor cells less susceptible to apoptosis and other damaging effects from ionizing radiation [89].

The response to radiation in the tissues surrounding tumors also follow daily circadian rhythms [52] and it might be possible to minimize side effects by administering radiotherapy to HGGs during a time of day when healthy tissues are less susceptible to its effects (chronoradiotherapy). Environmentally disrupted circadian rhythms in mice, brought on by a shifting light cycle, alters the gut bacterial composition and host radiosensitivity through an unexplained mechanism [90]. One study in breast cancer suggested that radiotherapy had more toxicity and side effects in the morning, and this was associated with a genetic variant of PER3 [91]. Time of treatment administration affected the response to radiation in females with bone metastases but had no effect in males, indicating that gender differences and sex hormones should be investigated for a role in circadian regulation of the response to radiotherapy [92]. A similar effect was seen in patients treated with whole brain radiotherapy for metastases. The time of day for whole brain radiotherapy was significantly related to overall survival in elderly females, while there was no apparent relationship in males [93]. Other clinical studies have shown that radiotherapy timing affects treatment response and toxicity depending on the tissue of interest [94–100], though some of them clearly had confounding factors. When these factors were accounted for, differences disappeared. For example, in one study, they found that patients with more severe signs of illness and metastases tended to be given afternoon appointments [96]. When this was accounted for, differences between groups disappeared.

Studies thus far on radiotherapy and circadian rhythms are limited by several factors. The studies conducted thus far are not directly comparable as the endpoints of individual trials are not the same. Some studies used overall survival as the endpoint, thus assessing circadian timing effects on tumor itself, while other studies used side effects as the endpoint, which generally reflects the effects of radiation on the surrounding healthy tissues. Healthy tissues may not have the same circadian oscillation as tumor tissues, and timing for maximal tumor response or for minimal healthy tissue damage therefore may not be the same, making it difficult to compare a small number of studies. Furthermore, there is no clear definition of morning, afternoon and evening, and the times used in different studies were not the same. No studies to our knowledge have focused on HGGs, and how hypoxia and circadian rhythms may interact to affect radiosensitivity of the tumor. Further prospective randomised control trials are required to determine if radiotherapy can be timed to target cancer cells and avoid toxicity in healthy tissues.

##### Circadian rhythms, metabolism and epigenetics

The circadian clock has strong links to metabolism, and cancer cells, including those in gliomas, have significantly altered metabolism that contributes to radioresistance. Specific diets and timed feedings can alter the circadian clock in various organs [101–106] and metabolites in human blood and muscle show rhythmic circadian patterns [107, 108], indicating there is a bidirectional relationship between circadian rhythms and metabolism. Animal models of jet lag and shift work can alter circadian rhythms, leading to increased rates of cancer and for some cancers this may be driven by metabolic changes linked to circadian disruption [109, 110]. The circadian clock also affects cellular metabolism and glioma cells are known to have abnormal metabolism.

Oncogenic changes in glioblastoma, including mutations in TP53, amplification of MYC or deletion of PTEN, alter metabolism and increase glycolysis [111, 112]. The expression of genes for MYC, PTEN and p53 are known to cycle in a circadian rhythmic fashion [83, 113] and there may be crosstalk between glioma cell metabolism and circadian rhythms. For example, in osteosarcoma and neuroblastoma cells, MYC disrupts the circadian clock by activating CLOCK and BMAL1, which alters glucose metabolism and glutaminolysis [114]. MYC has been shown to drive an abnormal metabolic program in glioblastoma cells and inhibition of this pathway shows therapeutic benefit in animal models [112]. Circadian rhythms can also act on the MYC pathway, as CRY2 promotes MYC degradation, and a loss of CRY2 stabilizes MYC, enhancing cellular transformation [115].

Additional HGG oncogenic pathways that control metabolism have been shown to interact bidirectionally with circadian rhythms. AKT and mTOR can set the pace for the circadian clock [116], and mTOR itself is cyclically ubiquitylated according to the clock [117]. While most of these findings have come from healthy humans/animals or cancers other than HGGs, one study found that GSCs can reprogram their metabolism to promote growth and survival and that this occurs through a mechanism using the circadian clock and epigenetic changes [11]. In GSCs, BMAL1 preferentially binds to the promoter region of a significant number of metabolism genes, particularly those involved in glycolysis and the TCA cycle. In normal neural stem cells, BMAL1 only bound to half of these genes. This indicates that BMAL1, a key circadian rhythm regulator, may be capable of inducing the expression of a significant number of metabolic genes specifically in GSCs [11]. The preferential binding was due to epigenetic histone modifications which enabled greater access to E-box binding sites in GSCs [11]. This study clearly demonstrates that circadian rhythms can be used by HGGs to alter metabolism, including glycolysis which supports HGG progression.

### Targeting HIF-1, glucose metabolism and circadian rhythms as an approach to improving radiosensitivity of HGGs

#### HIF-1

Theoretically, the deregulated glucose metabolism of tumor cells can be targeted at different levels, either by targeting the upstream transcriptional factor HIF-1 or via inhibiting key enzymes involved in downstream glucose metabolic pathways. HIF-1 inhibition leads to metabolic changes, with a decreased rate of glycolysis [118] and increased rate of tricarboxylic acid cycle [119]. Because the production of ROS are accompanied by enhanced mitochondrial oxidation, the therapeutic strategy of HIF-1 inhibition could potentially enhance the effect of radiotherapy [120], due to sharing the same mechanisms of cell killing. On the other hand, many other drugs targeting different signaling pathways may also be attributed partly to their secondary effect of HIF-1 inhibition. For example, anti-mitochondrial agents that reduce oxygen consumption spare more oxygen within tumor cells, leading to alleviated hypoxia and lower levels of HIF-1 [121]. Drugs targeting PI3K/Akt pathway also repress HIF-1 activation and sensitize tumor cells to apoptosis [122]. Although a large number of novel compounds have been shown to inhibit HIF-1 in preclinical setting, to the best of our knowledge there is no compound in clinical trials that directly and specifically inhibits HIF-1 activity. The molecular targeting of HIF-1 activity is very challenging because the pathways are complex, and it remains unclear where the main vulnerabilities lie.

As an alternative approach, hypoxia-activated prodrugs (HAPs) have been developed to specifically target hypoxic cells of tumors [123]. HAPs can be metabolized under hypoxic conditions to release an active drug that is more cytotoxic to hypoxic cells than normoxic cells through mechanism of bioreduction [124]. Therefore, the combination of radiotherapy and bioreductive drugs presents an attractive opportunity for synergistic effects. Nitro-based HAPs were the first generation of bioreductive drugs that undergo the oxygen-sensitive redox cycling [125]. This class of HAPs mimics oxygen to radiosensitize tumor cells [126]. Despite promising results from pre-clinical models, outcome of clinical trials were disappointing, with combination arm showing no significant survival benefit compared to radiotherapy alone in HGG patients [127]. The failure was thought to be due to the low drug concentrations achieved being not enough for radiosensitization [128]. Further attempts have been made to develop the second generation of HAPs including pimonidazole, etanidazole, and nimorazole. Due to the long half-lives of these drugs there was greater toxicity in human studies, and both pimonadazole and etanidazole showed negative results in phase I clinical trials [129], with nimorazole being the only one showing no major adverse effects in combination with radiotherapy [130]. It is now being routinely used in combination with conventional radiotherapy to treat head and neck cancers in Denmark [131] and being further evaluated in Phase II trials for the treatment of head and neck cancers in combination with hyperfractionated radiotherapy (NCT01880359, NCT01950689). Following the initial interest in oxygen mimetic bioreductive drugs, a new generation of DNA-targeting bioreductive prodrugs have been developed to be activated to cytotoxic drugs in the hypoxic environment, with PR-104 and TH-302 (evofosafamide) being the two representative drugs in this class. A recent preclinical study in breast cancer tumor xenografts indicated that both PR-104 and TH-302 sensitized tumors to irradiation, particularly in BRCA2-knockout mutants [132]. Although there are no clinical trials testing the two drugs in combination with radiotherapy, TH-302 has been reported to be safe in combination with VEGF inhibitor bevacizumab in patients with recurrent glioblastoma [133]. Tirapazamine (TPZ) is one of the best characterized HAPs that has been tested in various preclinical and early phase clinical trials and has yielded promising results. Preclinically, TPZ has showed synergistic or enhanced efficacy in combination with radiotherapy to treat melanoma cell lines and mouse models [134], which led to a phase 1 trial of TPZ and radiotherapy in the treatment of refractory solid tumors, suggesting TPZ can be safely used concurrently with radiotherapy as a radiosensitizer [135]. However, the combination of TPZ and chemoradiotherapy failed in a phase III trial for the treatment of head and neck cancers, although the hypoxic tumors were not selected upfront [131]. Moreover, the combination of TPZ and radiotherapy also failed to show benefit in a single-arm, open-labelled phase II study for glioblastoma. There was acceptable toxicity but no significant improvement in survival [136]. The repeated failings of trials testing these bioreductive prodrugs that have shown promising results preclinically raise questions as to which factors are limiting clinical success. Perhaps the most important limitation of bioreductive prodrugs in the context of these clinical trials is the failure to identify patients who are most likely to benefit from HAPs. Regarding the treatment of HGGs, penetration of the blood brain barrier is another critical factor that should be assessed comprehensively in both preclinical and clinical models.

#### Glucose metabolism

There are several key glycolytic enzymes that can be targeted pharmacologically in cancer treatment. The first orally administered small molecule that was tested in the early 1980’s to inhibit glycolysis was lonidamine, as an inhibitor of mitochondria-bounded hexokinase [137]. Lonidamine was later tested in a randomized clinical trial for glioblastoma in combination with radiotherapy, but failed to show therapeutic improvement compared to radiotherapy alone [138]. Following the failure of lonidamine, a structural analogue of glucose, 2-deoxy-D-glucose, was tested. Metabolically, it inhibits glycolysis via competitive inhibition of hexokinase 2 that controls the first rate-limiting step of glycolysis [139–141]. It was also reported to reduce the endoplasmic reticulum stress-related pathways which is significantly correlated to the radioresistance of GSCs [142]. Furthermore, 2-deoxy-D-glucose is also able to modify the DNA repair pathways to optimize radiotherapy in HGG treatment [143]. Treatment with 2-deoxy-D-glucose is cytostatic and radiosensitizes a range of cancer cells including HGGs [144]. These findings led to a phase I/II trials testing 2-deoxy-D-glucose in combination with radiotherapy in patients with HGG, which demonstrated this combined treatment was well tolerated without any acute toxicity or late radiation damage to the normal brain tissue [145]. This combination therapy also resulted in a moderate increase in median survival with a significantly improved quality of life [146]. Another leading compound 3-bromopyruvate, a pyruvate analogue, is both an alkylating agent and an inhibitor of hexokinase 2. 3-bromopyruvate inhibits tumor growth in a dose-dependent manner in vivo [147], but its off-target effect as well as the inability to penetrate the blood brain barrier impeded its further application in HGGs [148]. Another key enzyme that can be pharmacologically targeted is pyruvate dehydrogenase kinase, which controls the rate and amount of pyruvate entering the tricarboxylic acid cycle by negatively regulating pyruvate dehydrogenase activity. Inhibition of pyruvate dehydrogenase kinase shifts the glucose metabolism and increases oxygen consumption of tumor cells, which in turn inducing higher level of ROS, thus improving the radiosensitivity of the tumor cells [149]. Dichloroacetate (DCA), a small molecule inhibitor of pyruvate dehydrogenase kinase (PDK) with the potential for such metabolic modulation, has been shown to reverse the Warburg effect, thereby inhibiting both tumor cell growth and angiogenesis [150, 151]. By combining with radiotherapy, dichloroacetate enhances the radiosensitivity of several types of cancer in vitro [152–154] and in vivo including adult and pediatric HGGs [155, 156]. Dichloroacetate has been used as an orphan drug for various acquired and congenital disorders of mitochondrial metabolism in both adult and pediatric patients for decades and has recently been demonstrated to be feasible and well-tolerated in patients with recurrent HGGs in a recent Phase I clinical trial [157]. In addition, a recent study has tested the efficacy of DCA in a small cohort of HGG patients, suggesting metabolic modulation through pyruvate dehydrogenase kinase inhibition as a novel therapeutic strategy for the treatment of this devastating brain tumor [158]. Interestingly, the human toxicity from chronic DCA exposure is generally limited to a reversible peripheral neuropathy that is now known to be influenced by age. The dose-limiting neuropathy in the glioblastoma study above occurred at a DCA dose that is known to cause no side effects in children. The identical dose known to cause neuropathy in adults with mitochondrial disease has been safely given to children with congenital mitochondrial diseases for many years [159]. These findings and clinical results suggest that trials targeting the pediatric brain tumor population with DCA may be warranted, particularly given the safety and reduced toxicity of chronic administration of DCA in children. Moreover, melatonin, the secretory product of pineal gland, has also been recently reported to promote the synthesis of acetyl-CoA from pyruvate by inhibiting PDK in breast cancer models [160]. The inhibitory effect of melatonin on PDK not only reverses the Warburg effect, reduces tumor mass, and improves the sensitivity of tumor cells to chemoradiotherapy [160, 161], it also leads to a circadian rhythm of glucose metabolism in these cancer cells [162, 163].

#### Circadian clock

Because components of the molecular clock sustain growth, metabolism and stemness in HGGs [11], there may be opportunities to identify new drug targets and even combine with radiotherapy. Sulli et al., identified two REV-ERB agonists, SR9009 and SR9011, that impair glioblastoma growth in vivo and improve survival without causing toxicity [164]. REV-ERBs repress the transcription of BMAL1, so REV-ERB agonists should ultimately decrease BMAL1 levels. This would support the finding that BMAL1 drives growth and progression in glioblastoma [11]. The efficacy of SR9009 in a glioblastoma mouse model was similar to that of temozolomide, the current therapeutic standard for this cancer [164]. There are a number of other recently discovered compounds that target circadian proteins and some of these have been shown to have anti-cancer effects. Further work will be required to determine if this may be a useful strategy for inhibiting growth and improving the radiosensitivity of HGGs. On the other hand, future research has also been suggested to focus on chronoradiotherapy, where radiotherapy is given at specific times-of-the-day when a tumor is likely to be most susceptible to its effects and surrounding healthy tissues are the least susceptible [165]. Chronoradiotherapy may offer a promising new way to improve outcomes of cancer treatment as well as alleviating side-effects, though well-controlled studies are still needed to determine if there is a benefit. There are few prospective randomized control trials on chronoradiotherapy and only one study that has identified the genetic association between the time-of-day differences and radiotherapy outcomes [91]. Further work is also needed to identify a potential biomarker underlying this effect to predict which patients might benefit the most from chronoradiotherapy.

## Conclusions

**Fig. 4:**
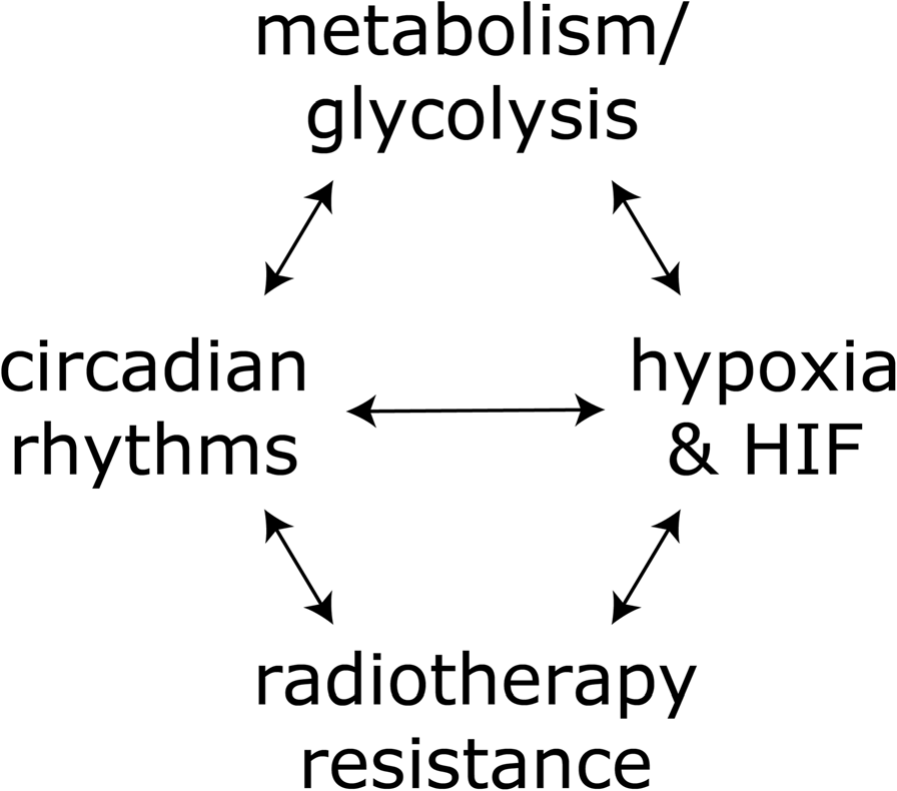
Interactions among hypoxia/HIF, tumor metabolism, circadian rhythm and radioresistance

HGGs are fast-growing, aggressive tumors with few treatment options. Radiotherapy is a first-line treatment option, though radioresistance commonly develops in HGGs and this is at least partially due to hypoxia and activation of the HIF pathway. HIF is also known to regulate the expression of genes involved in glycolysis and maintaining stemness of HGGs. Recent work has uncovered links between the circadian clock and HIF pathway while the circadian pathway has been found to drive proliferation, survival and stemness in GSCs [11, 70] (Fig. 4). Although there are a large number of studies focusing on all these abovementioned aspects, little is known about how HIF and circadian rhythms may interact on a mechanistic level, and how this interaction further modulates tumor metabolism and contributes to radioresistance in HGGs. The relationship between tumor metabolism, circadian rhythms and HIF is complex and future studies should focus on how these key pathways interact to affect the radiosensitivity of HGGs so that clinical outcomes can be further improved.

## Data Availability

Not applicable.

## Abbreviations

ADP: Adenosine diphosphate
ATP: Adenosine triphosphate
CA IX: Carbonic anhydrase 9
DCA: Dichloroacetate
DIPG: Diffuse intrinsic pontine glioma
G-6-P: Glucose-6-phosphate
G6PD: Glucose-6-phosphate dehydrogenase
GLUT: Glucose transporters
GSC: Glioma stem cell
GSH: Glutathione
GSSG: Glutathione disulfide
HAP: Hypoxia-activated prodrug
HGG: High-grade glioma
HIF-1: Hypoxia-inducible factor
HK: Hexokinase
LDHA: Lactate dehydrogenase A
LGG: Low-grade glioma
MCT4: Monocarboxylate transporter 4
NHE1: Na+/H+ exchanger isoform 1
PDH: Pyruvate dehydrogenase
PDK: Pyruvate dehydrogenase kinase
pHGG: Paediatric high-grade glioma
PPP: Pentose Phosphate Pathway
ROS: Reactive oxygen species
TIGAR: TP53-induced glycolysis and apoptosis regulator
TPZ: Tirapazamine
VEGF: Vascular endothelial growth factor
